# Trainable embedding quantum physics informed neural networks for solving nonlinear PDEs

**DOI:** 10.1038/s41598-025-02959-z

**Published:** 2025-05-29

**Authors:** Stefan Berger, Norbert Hosters, Matthias Möller

**Affiliations:** 1 Chair for Computational Analysis of Technical Systems, RWTH Aachen University, 52062 Aachen, Germany; 2 Department of Applied Mathematics, TU Delft, 2628 Delft, Netherlands

**Keywords:** Mathematics and computing, Computational science, Quantum physics

## Abstract

This paper proposes a novel approach for solving nonlinear partial differential equations (PDEs) with a quantum computer, the trainable embedding quantum physics informed neural network (TE-QPINN). We combine quantum machine learning (QML) with physics informed neural networks (PINNs) in a hybrid approach. By leveraging the advantages of classical and quantum computers, we can create algorithms that have a potential to be run on noisy intermediate-scale quantum devices (NISQ). We use feedforward neural networks (FNN) as problem-agnostic embedding functions, giving the used quantum circuit greater expressibility than previously introduced embedding. This expressibility allows us to solve a wide range of problems without using a problem specific ansatz. Additionally, we introduce a hybrid backpropagation algorithm that allows efficient updates of the used weights and biases in the FNN embedding functions. In this paper we showcase the capabilities of TE-QPINNs of a wide range of problems, including the two-dimensional Poisson, Burgers and Navier-Stokes equations. In direct comparison with classical PINNs, this approach showed an ability to achieve superior results while using the same number of parameters, highlighting their potential for more efficient optimization in high-dimensional parameter spaces, which could be transformative for future applications.

## Introduction

### Physics informed neural networks

**Fig. 1:**
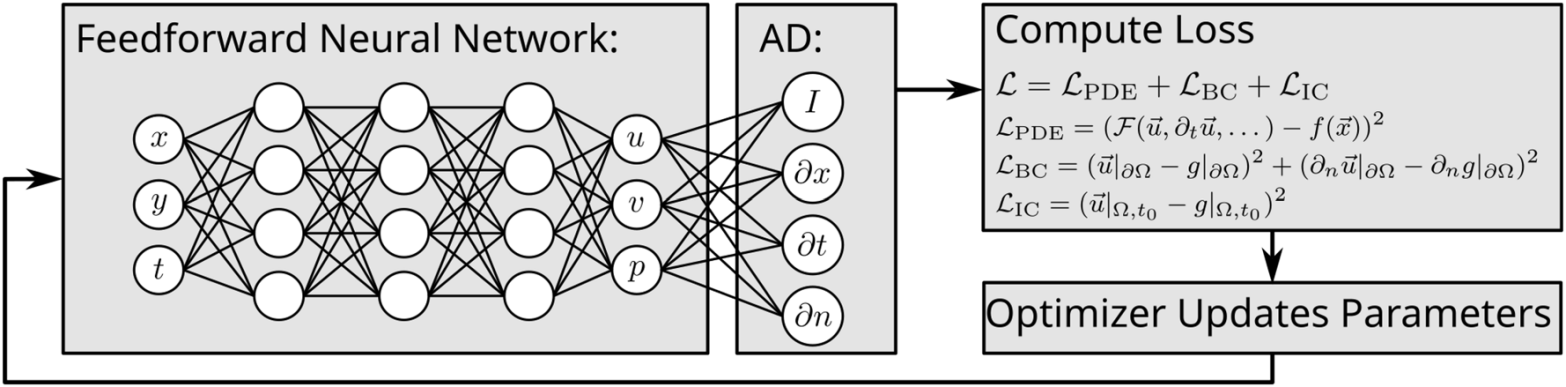
Schematic of a PINN. The FNN takes spatial and temporal inputs (*x*, *y*, *t*) and predicts the solution variables (*u*, *v*, *p*). Automatic differentiation (AD) is used to compute derivatives, which are then used to formulate the loss function incorporating PDE residuals, boundary conditions, and initial conditions. An optimizer updates the network parameters to minimize the loss and enforce the physics constraints.

Machine learning^1^ is a broad field that has seen significant growth in recent years and can be applied to a wide range of applications^2^. A subfield that has been of special interest is deep learning. Inspired by the human brain, neurons are packed into layers and are trained to process data. Many deep learning applications rely on the feedforward neural network (FNN). FNNs approximate functions that map an input to an output. They are built from layers. Each layer is typically vector-valued. Each entry in the vector can be interpreted as a “neuron”. The number of neurons is called the width of the layer. Each neuron has a number of trainable parameters, which are called weights and biases. Each neuron takes a vector input and outputs a scalar value. Non-linear activation functions can be applied after each layer to increase the function space that an FNN can represent. The number of layers is called the depth of the FNN. The parameters of the FNN need to be fitted for each new problem. An optimizer is trying to minimize a loss function by modifying the weights and biases (Fig. 1).

Physics-informed neural networks (PINNs)^3–5^ are a method to apply deep learning techniques to solve differential equations (DE). The goal is to train a model, usually an FNN, such that it approximates a solution to a DE within a domain $\Omega$. This is done by introducing a special loss function that is zero when the DE is perfectly solved. For this, the computational domain is sampled at so-called collocation points. The collocation points are divided into different groups, collocation points for each boundary, and collocation points within the domain $\Omega$. Each collocation point has a residual value. Inside the domain, this is the residual of a DE, or on the boundary it could be either a fixed value, corresponding to a Dirichlet boundary condition, or a derivative w.r.t. to the input variable, corresponding to a Neumann boundary. For the loss function, we iterate over all collocation points and sum the residual values. An optimizer is used to train parameters of the FNN in order to minimize the loss function. PINNs aim to harness the advancements in deep learning and apply them to engineering problems. However, training PINNs presents significant challenges due to the need to find a global minimum in a high-dimensional parameter space (For more details, see the optimization section in the review paper^5^). This global minimum is crucial because it represents the point at which the residual of the DE approaches zero, potentially yielding a solution to the problem. This stands in stark contrast to traditional machine learning tasks, such as image classification between cats and dogs, where a local minimum achieving 95% accuracy might be considered satisfactory. In the context of PINNs, such a local minimum would likely lead to physically inconsistent or unrealistic solutions, underscoring the unique challenges in training these networks for scientific and engineering applications.

### Quantum machine learning

Quantum mechanics is a theory in physics that describes nature at the levels of atoms and subatomic particles. Unlike classical mechanics, which offers deterministic predictions, quantum mechanics is inherently probabilistic. Quantum computing uses principles from the quantum world like superposition and entanglement for solving problems. By using those principles that are not available to classical hardware, better scaling for certain kinds of problems can be achieved. A prime example of this is the Shor algorithm^6^ for factoring large integers. The algorithm provides an exponential speedup compared to the best-known classical algorithms. This sounds very promising, however, currently no quantum computer exists that runs algorithms such that one can benefit from the scaling. A critical challenge in constructing quantum computers lies in the inherent fragility of quantum states. Since perfect isolation is unachievable, quantum states inevitably become entangled with their environment in a phenomenon known as decoherence. Decoherence introduces unpredictable and uncontrollable alterations to the quantum state. Consequently, current devices are classified as noisy intermediate-scale quantum devices (NISQ), and are unsuitable for running many proposed quantum algorithms due to operational and measurement noise^7^.

While single bits are the fundamental building block of classical computing, qubits are the corresponding analog of quantum computing. Single bits are limited to states of either 0 and 1. A qubit has $\mathinner {|{0}\rangle }$ and $\mathinner {|{1}\rangle }$ as the computational basis states. The first big difference between qubits and classical bits is that a qubit can be in a linear combination of its basis states. When this is the case, we talk about a superposition:

1 $$\begin{aligned} \mathinner {|{\psi }\rangle } = \alpha \mathinner {|{0}\rangle } + \beta \mathinner {|{1}\rangle } \quad \text {, where } \alpha , \beta \in \mathbb {C}. \end{aligned}$$

All information about a quantum system is encoded in the basis vectors and the amplitudes $\alpha$ and $\beta$. We can easily read the state of a classical bit. Reading a quantum state is called a measurement. When measuring a quantum state, we perform a probabilistic operation. The possible states that we could measure are $\mathinner {|{0}\rangle }$ or $\mathinner {|{1}\rangle }$. The chance of measuring $\mathinner {|{0}\rangle }$ or $\mathinner {|{1}\rangle }$ is $|\alpha |^2$ and $|\beta |^2$, respectively. The total probability should sum up to 1:

2 $$\begin{aligned} |\alpha |^2 + |\beta |^2 = 1. \end{aligned}$$

**Fig. 2:**
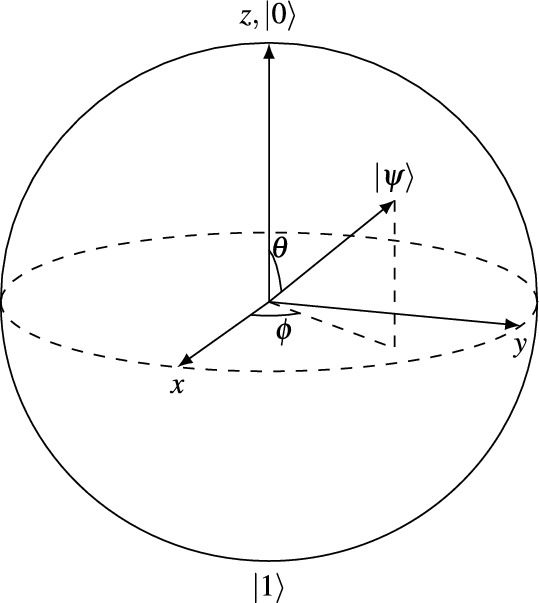
Illustration of the Bloch sphere, where each location on the sphere represents a feasible qubit state. The north and south poles correspond to the computational basis states $\mathinner {|{0}\rangle }$ and $\mathinner {|{1}\rangle }$, respectively. The vector $\mathinner {|{\psi }\rangle }$ is depicted within the sphere. Upon measurement, the state vector will align itself either towards the north or south pole, depending on the measurement outcome.

After the measurement, the qubit collapses to the measured state. A tool to visualize a single qubit is the so-called Bloch sphere, which is shown in Fig. 2. We can rewrite Eq. (1) as:

3 $$\begin{aligned} \mathinner {|{\psi }\rangle } = \cos \left( \frac{\theta }{2}\right) \mathinner {|{0}\rangle } + e^{i\phi } \sin \left( \frac{\theta }{2}\right) \mathinner {|{1}\rangle }, \end{aligned}$$

where $\theta$ and $\gamma$ are real numbers.

Now we assume that we have more than one bit. In the case of a classical two-bit configuration, this would yield the four potential states: 00, 01, 10, and 11. The basis vectors for the quantum system look similar: $\mathinner {|{00}\rangle }$, $\mathinner {|{01}\rangle }$, $\mathinner {|{10}\rangle }$ and $\mathinner {|{11}\rangle }$. This state can be constructed using the tensor product on the basis of the states of each subsystem $\{\mathinner {|{0}\rangle }, \mathinner {|{1}\rangle } \} \otimes \{\mathinner {|{0}\rangle }, \mathinner {|{1}\rangle } \} =\{\mathinner {|{00}\rangle },\mathinner {|{01}\rangle }, \mathinner {|{10}\rangle },\mathinner {|{11}\rangle }\}$. Again, each basis vector has a complex coefficient, which is also called the amplitudes. All possible states for a system of two qubits can be stated like this:

4 $$\begin{aligned} \mathinner {|{\psi }\rangle } = a_{00}\mathinner {|{00}\rangle } + a_{01}\mathinner {|{01}\rangle } + a_{10}\mathinner {|{10}\rangle } + a_{11}\mathinner {|{11}\rangle } \quad \text {, where } a_{i} \in \mathbb {C} . \end{aligned}$$

To avoid breaking physics, the sum of probabilities should add up to 1. A famous two-qubit state is the Bell state:

5 $$\begin{aligned} \mathinner {|{\phi ^*}\rangle } = \frac{\mathinner {|{00}\rangle } +\mathinner {|{11}\rangle }}{\sqrt{2}}. \end{aligned}$$

Observing that the coefficients for $\mathinner {|{01}\rangle }$ and $\mathinner {|{10}\rangle }$ are zero, hence they are omitted in the expression. This quantum state exhibits a 50% probability of measuring either 0 or 1 upon assessing the first qubit. Post-measurement, the state reduces to $\mathinner {|{00}\rangle }$ if the first qubit is found to be $\mathinner {|{0}\rangle }$, or to $\mathinner {|{11}\rangle }$ if it is $\mathinner {|{1}\rangle }$. Consequently, the second qubit is now correlated with the observed state of the first qubit. This phenomenon, termed entanglement, augments the superposition principle and stands as a fundamental concept in quantum computing and information theory. Entanglement facilitates the exploration of numerous intriguing quantum phenomena, including quantum teleportation. A system consisting of *n* qubits can be expressed as $\mathinner {|{x_{1}x_{2}\ldots x_{n}}\rangle }$, with this state comprising $2^n$ amplitudes. When using 500 qubits, it results in $2^{500}$ coefficients, a quantity exceeding the estimated number of atoms in the universe, rendering it unfeasible to store these many values using classical computing methods.

We will describe quantum computing in the circuit model, where operations are modeled as elementary quantum gates. Algorithms are implemented by applying a series of quantum gates to the qubit register. Quantum gates could implement any unitary transformation. For this work the parameterized rotation gates and the CNOT gate are important. The parameterized rotation gates are operations that only act on a single qubit. They rotate the qubit state around one of the coordinate axes. This can be nicely visualized on the Bloch sphere. The CNOT gate acts on two qubits where one acts a control and the other one as the target. This means that if the control qubit is $\mathinner {|{1}\rangle }$, the amplitudes of the target qubit will be flipped. When the control qubit is a superposition between $\mathinner {|{0}\rangle }$ and $\mathinner {|{1}\rangle }$, it will lead to a superposition of applied and nonapplied states.

A class of methods that was designed for NISQ devices are variational quantum algorithms (VQA). VQAs have shown great promise in many fields^8^ including quantum chemistry, combinatorial optimization, and machine learning. The key to VQAs recent success is the fact that the workload of the task is split to be partly computed on a quantum computer and a classical computer. The goal is to minimize a cost function $C(\theta )$ that depends on a variation ansatz with parameters $\theta$. The cost function encodes the problem and is evaluated on the quantum computer; after that, the variational parameters are updated using an optimization algorithm that runs on a classical computer. This is called a hybrid approach. Using both computing frameworks, we can take advantage of their individual strengths.

**Fig. 3:**
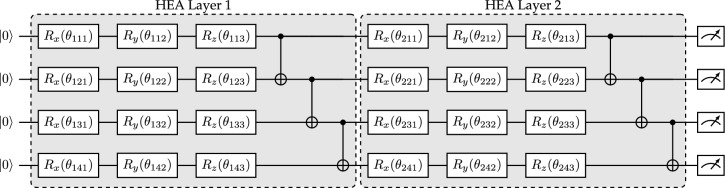
Four qubit quantum circuit implementing the hardware-efficient ansatz (HEA). It utilizes two layers, where each qubit gets rotated around each axis of the Bloch sphere before being entangled with its lower and upper neighbor using CNOT gates. The arrangement of CNOT gates and number of rotational gates varies in literature. The initial quantum register is initialized as $\mathinner {|{0}\rangle }$. After the all gates have been performed, every qubit is measured.

Building on top of VQA is Quantum Machine Learning (QML). QML shares many similarities with VQAs, and the insights gained from one class of algorithms often apply to the other. However, QML introduces two key modifications to VQAs: it allows for input data and incorporates a loss function, making it closely resemble classical machine learning. In VQAs, the primary goal is to find a quantum state that minimizes a given cost function. In QML, the objective is to train a quantum circuit so that for a set of data inputs, the expectation value of the quantum circuit reduces a loss function. The role of the quantum circuit in this context is analogous to that of a FNN in classical machine learning. Both are designed to act as parameterized function approximators. A quantum circuit utilizing the hardware efficient ansatz (HEA) (Fig. 3) has been shown to operate as a universal function approximator, similar to an FNN, as long as a sufficient number of layers and qubits are employed^9^.

**Fig. 4:**
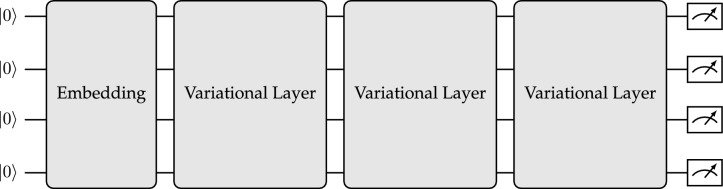
Potential configuration for a parameterized quantum circuit that can be used in quantum machine learning. Initially, the embedding is applied, which incorporates the input value into the quantum state. Following this, the variational ansatz is implemented. In this illustration, three layers of a hardware-efficient ansatz are utilized. Finally, the cost function value is computed.

Differential circuits have been proposed as a method to solve DE on quantum processing units (QPU)^10,11^. They use a parameterized quantum circuit (PQC) (Fig. 4) instead of an FNN and use the parameter shift method instead of algorithmic differentiation for the derivatives of the collocation points. The parameter-shift method allows the computation of derivatives of the expectation value of a PQC w.r.t. the values that parameterize the quantum circuit^12–14^. Building on top of differential circuits, this work will introduce the trainable embedding quantum physics informed neural network (TE-QPINN).

## TE-QPINNs

TE-QPINNs are a method for solving differential equations using ideas from quantum computing and deep learning. TE-QPINN stands for trainable embedding QPINN. It is an evolution of the differentiable circuits introduced by Kyriienko et al. in^11^. The authors of the paper stated in their conclusion that the goal is to find quantum feature maps that can efficiently represent the target function. We will later see that their proposed method of using their Chebyshev polynomial embedding works well when you have enough qubits available and the target function is smooth enough. Since the number of usable qubits is currently limited, they concluded that a good choice of the quantum feature map is problem-specific. We propose a method where the quantum feature map is a trained FNN. This paper will show that this has multiple benefits.

1. This method is problem-independent and can solve a wide range of problems.
2. The FNN embedding approach is more efficient than the polynomial approach used in^11^, in the sense that it can deliver better results for the same number of qubits
3. By leaning more towards the hybrid approach, which is classical and quantum computing combined, we can drastically reduce the computational effort to train the FNN parameters.

The first point is achieved through the use of an FNN. As shown by the universal approximation theorem, FNN can approximate almost any function, making them highly adaptable. The second point will be illustrated across a broad set of examples. The final point is premised on the ability to compute the gradients of the loss w.r.t. the FNN parameters on classical hardware, with partial dependence on the parameter shift rule. This will be verified using the chain rule. Furthermore, as the examples will demonstrate, a relatively small FNN can be sufficient to achieve satisfactory results.

The TE-QPINN has four main components: the embedding function, the variational ansatz, the cost function and the loss function. In the following, each of the component will be explained in detail. Following that, we explain how to train a TE-QPINN.

The general differential equation problem to be solved later on can be stated as follows:

6 $$\begin{aligned} \mathscr {F}(u(x); \gamma )&= f(x) \quad x \in \Omega , \end{aligned}$$

7 $$\begin{aligned} \mathscr {B}(u(x))&= g(x) \quad x\in \partial \Omega , \end{aligned}$$

where:

- $\Omega \subset \mathbb {R}^{d}$ is the domain with the boundary $\partial \Omega$,
- $x \in \mathbb {R}^d$ is the space-time coordinate vector,
- *u* is the unknown field,
- $\mathscr {F}$ is a non-linear differential operator with parameters $\gamma$ and *f* being the physical data
- $\mathscr {B}$ is an operator to assign values to the boundary, with *g* being a function on the boundary. Boundary conditions can be Dirichlet, Neumann, Robin, or periodic.

The general quantum circuit can be written as:

8 $$\begin{aligned} \tilde{u}(x; \theta ,\Xi ) = C(x,\theta ,\Xi ) = \sum _{i=0}^n \mathinner {\langle {0 | U_{\text {emb}}(x, \Xi )^\dagger U_{\text {var}}(\theta )^\dagger O U_{\text {var}}(\theta )U_{\text {emb}}(x,\Xi )}|}\mathinner {|{0}\rangle }, \end{aligned}$$

where:

- $C(x,\theta ,\Xi )$ is the expectation value of the circuit, which is used as the approximation $\tilde{u}$ of the unknown field *u*,
- *n* is the count of qubits or wires in the circuit,
- $U_{\text {emb}}(x, \Xi )$ is the operation that embeds the state-space vector *x* into the quantum state, $\Xi$ are the weights and biases of the underlying FNN model that is used to compute the values of the angle embedding,
- $U_{\text {var}}(\theta )$ is the operation performed by the variational ansatz which is parameterized by $\theta$.

*Embedding* The embedding of the TE-QPINN is the essential cornerstone of the method. It is called trainable embedding because we utilize a trainable model to find a suitable way to embed the classical state *x* into the quantum state of the quantum circuit. The results shown in this paper utilize an FNN; however, any trainable model should work. The embedding is performed using angle embedding, meaning that qubits are rotated by an angle around a single axis, for this work we choose the *y*-axis. The FNN is used as the quantum feature map $\Phi$ that maps the input *x* to a scaling factor $\phi _i(x)$ for each qubit. The final rotation angle is given by $\phi _i(x) \cdot x$. During testing, we found that $\phi _i(x) \cdot x$ performed better than just $\phi _i(x)$.

The steps to perform the embedding are as follows. First, rescale the data set to ensure that all features are equally weighted. We rescale between $x_\text {min}$ and $x_\text {max}$.

9 $$\begin{aligned} \tilde{x} = \frac{x - x_\text {min}}{x_\text {max} - x_\text {min}} \quad \text {s.t.} \ x_\text {min} \le \tilde{x} \le x_\text {max}. \end{aligned}$$

After that, the FNN model is evaluated at $\tilde{x}$.

10 $$\begin{aligned} \Phi (\tilde{x}) = \begin{pmatrix} \phi _1(\tilde{x})&\phi _2(\tilde{x})&\ldots&\phi _n(\tilde{x}) \end{pmatrix}^T \quad \text {, where }n\text { is the number of qubits in the circuit.} \end{aligned}$$

This results in a scaling factor $\phi _i(\tilde{x})$ for every qubit which can be used to apply the desired angles. For the embedding, we only rotated around the y-axis.

11 $$\begin{aligned} U_\text {emb}(x) = \bigotimes _{i=0}^{n} \, R_y^i(\phi _i(\tilde{x}) \cdot \tilde{x}) \end{aligned}$$

When solving PDEs where *x* is two-dimensional or higher, an iterative cycle through the components of *x* using the modulus operation $i \% n$ is applied. The number of qubits should be divisible by the number of dimensions. For example, with 4 qubits and $x \in \mathbb {R}^2$, the transformations to the components in the form $(\phi _1 x_1, \phi _2 x_2, \phi _3 x_1, \phi _4 x_2)$ can be applied.

*Variational ansatz* The variational ansatz $U_\text {var}(\theta )$ evolves the quantum state, with its parameters being optimized using the LBFGS optimizer^15^, so that the measurement of the state provides a good approximation of the unknown field *u*. Here, the HEA that was already shown in Fig. 3 will be utilized. The HEA has two benefits that work to our advantage. First, one can easily add more layers to make the circuit more expressive and keep the count of needed gates to a minimum. This makes it promising to be implemented on a real quantum computer in the near future. Second, the variational ansatz can be written as a series of unitary transformations:

12 $$\begin{aligned} U_{\text {var}}(\theta ) = U_L(\theta _{L})\cdots U_2(\theta _2) U_1(\theta _1), \quad \text {with} \quad U_l(\theta _l) = \prod _{m} e^{-i\theta _{l,m} H_{m}} W_m, \end{aligned}$$

where $W_m$ is an unparameterized unitary, and $H_{m}$ is a Hermitian operator. Each $U_l(\theta _l)$ is a layer of the ansatz which is a product of all gates (denoted with *m*). A layer consists of rotation gates followed by a chain of CNOT operations. The rotation gates used in this work are $R_x$, $R_y$, and $R_z$ for each qubit.

*Cost function* The cost function $C(x; \theta , \Xi )$ takes the quantum state generated by the embedding and the variational ansatz and returns a measurement result of the associated observable *O*. A common observable is:

13 $$\begin{aligned} O = \bigotimes _{i=1}^n Z_i. \end{aligned}$$

It measures the state of every qubit along the computational basis and sums the resulting measurements. The choice of the cost function has a significant influence on the convergence of the optimizer and the cost landscape, as shown in^16^. When working with a quantum state simulator, the expectation value of the observable can directly be computed. This is not possible on quantum hardware, there we need to evaluate the quantum circuit multiple times to get an estimate of the expectation value.

*Loss function* The loss function $\mathscr {L}(\theta ,\Xi )$ in the TE-QPINN method is identical to that used in PINNs. You have sets of collocation points that correspond either to the interior of the domain $\Omega$ or one of the boundaries $\partial \Omega _j$. Let $\mathscr {I}_\text {PDE}$ and $\mathscr {I}_{\text {BC},j}$ be the set of collocation points, we then define the loss function as:

14 $$\begin{aligned} \mathscr {L}(\theta ,\Xi )&= \mathscr {L}_\text {PDE}(\theta ,\Xi ) + \sum _k \lambda _k \mathscr {L}_{\text {BC},k}(\theta ,\Xi ) \end{aligned}$$

15 $$\begin{aligned} \mathscr {L}_\text {PDE}&= \sum _{x^j\in \mathscr {I}_\text {PDE}} \, \left( \mathscr {F}(\tilde{u}(x^j); \gamma ) - f(x^j)\right) ^2 \end{aligned}$$

16 $$\begin{aligned} \mathscr {L}_{\text {BC},k}&= \sum _{x^j \in \mathscr {I}_{\text {BC},k}} \, \left( \mathscr {B}_k(\tilde{u}(x^ij) - g(x^j)\right) ^2 \end{aligned}$$

Initial conditions are treated as temporal boundary values of the space-time domain. Each boundary is individually weighted with a scalar factor $\lambda _j$. A high value will make the optimizer focus on that region since the gradient will be larger. This is especially useful for the initial condition since it has to be upheld, otherwise a different initial value problem is solved.

For the TE-QPINN, two types of derivatives are required. First, to evaluate the residual of the differential equation at the collocation points within the domain, derivatives of the cost function $C(x; \theta , \Xi )$ w.r.t. *x* are needed. Second, for gradient-based optimization, derivatives of the loss function $\mathscr {L}( \theta , \Xi )$ w.r.t the model parameters $\theta$ and $\Xi$ are needed to update the latter. The unmodified parameter-shift rule^12,14^ could be used to compute those derivatives. However, applying this method individually to each parameter in an FNN with numerous trainable parameters would require many quantum circuit evaluations, scaling with the FNN’s parameter count. This process can be optimized by combining the parameter-shift rule with the backpropagation algorithm. This hybrid approach reduces the required circuit evaluations to twice the number of qubits in the quantum circuit, independent of the FNN size. The chain rule allows us to split the derivative into two components: the quantum circuit derivative (evaluated via the parameter shift rule) and the FNN derivative (computed through backpropagation). The quantum circuit’s partial derivative remains constant and can be reused, while the FNN’s partial derivatives are efficiently calculated on a classical computer.

The derivatives of the cost function $C(x; \theta , \Xi )$ can be written as follows:

17 $$\begin{aligned} \frac{\partial \tilde{u}}{\partial x}=\frac{\partial C}{\partial x} = \sum _{i=1}^{n} \left( \underbrace{\frac{\partial C}{\partial R_y^i} \cdot \frac{\partial R_y^i}{\partial (\phi _i(x) \, x)}}_{\text {parameter-shift rule}} \cdot \left( 1 + \underbrace{\frac{\partial \phi _i(x)}{\partial x}x}_{\text {backpropagation}} \right) \right) \end{aligned}$$

Since the qubits are rotated by $\phi _i(x) \cdot x_i$, the product rule to get the accurate derivates has to be utilized.

The derivatives of the loss function $\mathscr {L}( \theta , \Xi )$ w.r.t. the FNN model parameters $\Xi$ can be stated as follows:

18 $$\begin{aligned} \frac{\partial \mathscr {L}}{\partial \Xi }&= \frac{\partial \mathscr {L}_\text {PDE}}{\partial \Xi } + \sum _k \frac{\partial \mathscr {L} _{\text {BC},k}}{\partial \Xi } \end{aligned}$$

19 $$\begin{aligned} \frac{\partial \mathscr {L}_\text {PDE}}{\partial \Xi }&= \frac{\partial }{\partial \Xi } \sum _{x^jin \mathscr {I}_\text {PDE}} \, \left( \mathscr {F}(\tilde{u}(x^j) - f(x^j) \right) ^2 \end{aligned}$$

20 $$\begin{aligned}&= 2 \sum _{x^j\in \mathscr {I}_\text {PDE}} \, \frac{\partial }{\partial \Xi } \left( \mathscr {F}(\tilde{u}(x^j)) - f(x^j) \right) \end{aligned}$$

21 $$\begin{aligned}&= 2 \sum _{x^j\in \mathscr {I}_\text {PDE}} \, \frac{\partial \mathscr {F}(\tilde{u}(x^j))}{\partial \Xi } \end{aligned}$$

22 $$\begin{aligned}&= 2 \sum _{x^j\in \mathscr {I}_\text {PDE}} \, \frac{\partial \mathscr {F}(\tilde{u}(x^j))}{\partial \tilde{u}} \frac{\partial \tilde{u}}{\partial \Xi } \end{aligned}$$

23 $$\begin{aligned}&= 2 \sum _{x^j\in \mathscr {I}_\text {PDE}} \, \frac{\partial \mathscr {F}(\tilde{u}(x^j))}{\partial \tilde{u}} \sum _{i=0}^n \left( \underbrace{\frac{\partial C}{\partial R_y^i} \cdot \frac{\partial R_y^i}{\partial (\phi _i(x^j) \, x^j)}}_{\text {parameter-shift rule}} \cdot \underbrace{\frac{\partial \phi _i(x^j)}{\partial \Xi } x^j}_{\text {backpropagation}} \right) \end{aligned}$$

24 $$\begin{aligned} \frac{\partial \mathscr {L}_{\text {BC},k}}{\partial \Xi }&= \frac{\partial }{\partial \Xi } \lambda _k \sum _{x^j \in \mathscr {I}_{\text {BC},k}} \, \left( \mathscr {B}_k(\tilde{u}(x^j) - g(x^j) \right) ^2 \end{aligned}$$

25 $$\begin{aligned}&= 2 \lambda _k \sum _{x^j \in \mathscr {I}_{\text {BC},k}} \, \frac{\partial }{\partial \Xi } \left( \mathscr {B}_k(\tilde{u}(x^j)) - g(x^j) \right) \end{aligned}$$

26 $$\begin{aligned}&= 2 \lambda _k \sum _{x^j \in \mathscr {I}_{\text {BC},k}} \, \frac{\partial \mathscr {B}_k(\tilde{u}(x^j))}{\partial \Xi } \end{aligned}$$

27 $$\begin{aligned}&= 2 \lambda _k \sum _{x^j \in \mathscr {I}_{\text {BC},k}} \, \frac{\partial \mathscr {B}_k(\tilde{u}(x^j))}{\partial \tilde{u}} \frac{\partial \tilde{u}}{\partial \Xi } \end{aligned}$$

28 $$\begin{aligned}&= 2 \lambda _k \sum _{x^j \in \mathscr {I}_{\text {BC},k}} \, \frac{\partial \mathscr {B}_k(\tilde{u}(x^j))}{\partial \tilde{u}} \sum _{i=0}^n \left( \underbrace{\frac{\partial C}{\partial R_y^i} \cdot \frac{\partial R_y^i}{\partial (\phi _i(x^j) \, x^j)}}_{\text {parameter-shift rule}} \cdot \underbrace{\frac{\partial \phi _i(x^j)}{\partial \Xi } x^j}_{\text {backpropagation}} \right) \end{aligned}$$

### Hybrid training loop

**Fig. 5:**
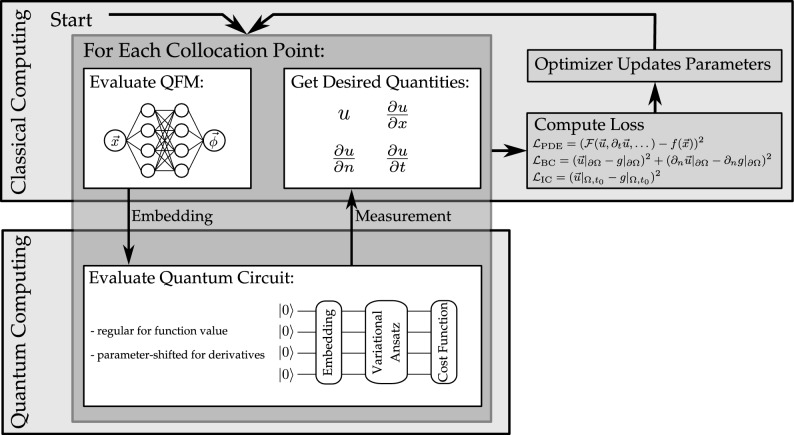
The hybrid training loop employed to train TE-QPINNs.

The training of the TE-QPINN works as follows. First, the quantum circuit and the problem need to be initialized. After that, the variational parameters are optimized in a training loop. The training loop is illustrated in Fig. 5.

*Initialization* The initialization process for the TE-QPINN algorithm begins by defining the loss function. This involves specifying the function and boundary operator, as well as discretizing the domain into collocation points. Next, we establish the quantum computing architecture, which includes choosing the structure of the embedding FNN by choosing its depth and width. We then select a variational ansatz with trainable parameters, decide on the number of layers, and choose an appropriate observable for the cost function. At this stage, we also initialize the parameters. The final step in our initialization is defining an optimizer. In general, we recommend using the L-BFGS due to its effectiveness in handling complex optimization landscapes.

*Training loop* The TE-QPINN training loop iterates until the loss function is sufficiently small. Each iteration involves:

1. Evaluating the TE-QPINN at collocation points to approximate function values.
2. Computing derivatives for loss function residuals using a combination of the parameter-shift rule and backpropagation.
3. Updating the FNN and variational ansatz parameters with an optimizer.

## Results

We will now benchmark the TE-QPINN method through a sequence of problems. The quantum circuits are implemented using the PennyLane framework^17^. We utilize a quantum state simulator that computes the exact expectation value of the PCQ. The FNNs are constructed using PyTorch^18^. Each FNN is composed of an input layer that receives the dimension corresponding to the computational domain, succeeded by several hidden layers. Ultimately, an output layer generates a vector with dimensions equal to the number of qubits. The TanH activation function is used after the input and each hidden layer. All models are trained using the L-BFGS optimizer as implemented in PyTorch using the default parameter with the strong Wolfe conditions. The code for all shown examples can be found in the following git repository: https://git.rwth-aachen.de/berger.st.11.11/te-qpinns#.

### ODE equation

The first test case originates from the differential circuit paper^11^. Here, the Chebyshev embedding and the Tower-Chebyshev embedding are now compared to the TE-QPINN. The differential equation is given by:

29 $$\begin{aligned} \frac{du}{dx}&= 4u - 6u^{2} + \sin (50x) + u\cos (25x) - 0.5, \quad x\in [0,1] \quad \text {and} \quad u(0)=0.75. \end{aligned}$$

For this experiment, 100 collocation points are uniformly spaced across the domain. The initial condition at $x=0$ is given a scaling factor of 10, and the input coordinate *x* is rescaled to $[-0.95, 0.95]$. We obtain our reference solution using the Runge-Kutta 45 method. The TE-QPINN employs an FNN with just a single hidden layer with five neurons.

**Fig. 6:**
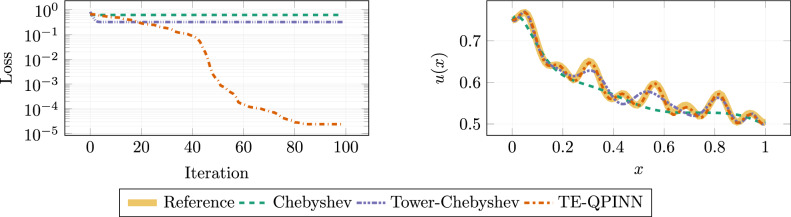
The graph on the left shows the loss history for the different methods. On the right, the reference solution as well as functions computed by the different embeddings are shown. The quantum circuits in these methods consist of four qubits and five variational layers.

Various configurations of variational layers and qubits have been explored. The results of these configurations are shown in Table 1 for the Tower-Chebyshev embedding and in 2 for the TE-QPINN. Each setup was executed three times with varied initial parameters and, subsequently, the average loss was calculated. Outcomes for four qubits and five variational layers are presented in Fig. 6.

It can be observed that the Tower-Chebyshev embedding is unable to converge for two or four qubits. The method gets stuck at the same minimum, regardless of the number of variational layers used. When using six or eight qubits, the loss function decreases when increasing the number of variational layers to 5. Using more than five layers does not yield an additional benefit. Switching to the TE-QPINN in Table 2, the method does not converge for two qubits; however, it does converge for four, six, and eight. Four qubits require three layers or more to obtain a loss lower than $10^{-4}$. When using six or eight qubits, a single variational layer is enough.

In Fig. 6 we can see that only the TE-QPINN is capable of capturing the full solution with high accuracy when using four qubits and five variational layers. The Chebyshev embedding is only able to capture the general trend of the solution. An upgrade to the Tower-Chebyshev yields some improvement but is also not able to capture all solutionn details. In contrast, TE-QPINNs can achieve a crips resolution with the same qubit count.

**Table 1:** Loss values for different combinations of Layers and Qubits using the Tower-Chebyshev quantum feature map.

Layers	Qubits			
	2	4	6	8
1	$1.27\times 10^{1}$	7.56	7.05	5.83
3	5.16	$3.30\times 10^{-1}$	$2.83\times 10^{-3}$	$2.88\times 10^{-3}$
5	5.16	$3.20\times 10^{-1}$	$6.38\times 10^{-6}$	$3.99\times 10^{-6}$
7	5.16	$3.20\times 10^{-1}$	$2.12\times 10^{-5}$	$6.57\times 10^{-6}$
10	5.16	$3.20\times 10^{-1}$	$9.54\times 10^{-6}$	$1.60\times 10^{-5}$

**Table 2:** Loss values for different combinations of Layers and Qubits using the TE-QPINN with one hidden layer and five neurons each.

Layers	Qubits			
	2	4	6	8
1	$1.95\times 10^{-1}$	$8.92\times 10^{-2}$	$3.92\times 10^{-5}$	$1.75\times 10^{-5}$
3	$8.32\times 10^{-2}$	$2.80\times 10^{-5}$	$1.02\times 10^{-5}$	$4.26\times 10^{-5}$
5	$1.92\times 10^{-1}$	$1.52\times 10^{-4}$	$2.50\times 10^{-5}$	$2.39\times 10^{-5}$
7	$2.90\times 10^{-1}$	$1.31\times 10^{-4}$	$1.98\times 10^{-5}$	$2.85\times 10^{-5}$
10	$1.48\times 10^{-1}$	$1.50\times 10^{-4}$	$1.46\times 10^{-5}$	$9.38\times 10^{-6}$

### PDE equations

In this section, more complex PDE problems are investigated.

**Fig. 7:**
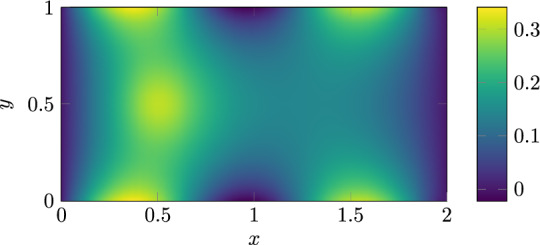
Reference solution to the Poisson equation 30.

**Poisson equation** We benchmarked the Poisson equation with boundary and source terms taken from an example on the Fenics project website^19^. The PDE has both Dirichlet and Neumann boundary conditions and a source term. The reference solution of the field *u* can be seen in Fig. 7. It was calculated using the Fenics finite element solver. We will compare the TE-QPINN with the Tower-Chebyshev embedding. The problem is stated as follows:

30 $$\begin{aligned} - \Delta u&= f \quad \text {in } \Omega , \end{aligned}$$

31 $$\begin{aligned} u&= 0 \quad \text {on } \Gamma _D,\end{aligned}$$

32 $$\begin{aligned} \nabla u \cdot n&= g \quad \text {on } \Gamma _N, \end{aligned}$$

with:

33 $$\begin{aligned} \Omega&=[0,2]\times [0,1], \end{aligned}$$

34 $$\begin{aligned} \Gamma _{D}&= \{(0, y) \cup (2, y) \subset \partial \Omega \} \end{aligned}$$

35 $$\begin{aligned} \Gamma _{N}&= \{(x, 0) \cup (x, 1) \subset \partial \Omega \} \end{aligned}$$

36 $$\begin{aligned} g&= \sin (5x) \end{aligned}$$

37 $$\begin{aligned} f&= 10\exp (-((x-0.5)^2 + (y-0.5)^2)/0.02). \end{aligned}$$

The domain was discretized with 50 evenly distributed points in the x direction and 25 points in the y direction. Each dimension of the input data was rescaled to the range [-0.95, 0.95]. A scaling of 10 is applied to all boundaries. The FNN in the TE-QPINN utilizes two hidden layers with ten neurons each.

**Fig. 8:**
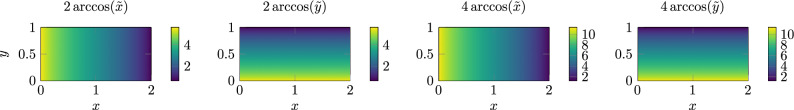
The rotation angles for the Tower-Chebyshev feature map applied to the rescaled coordinates. They stay the same, independent of the problem.

**Fig. 9:**
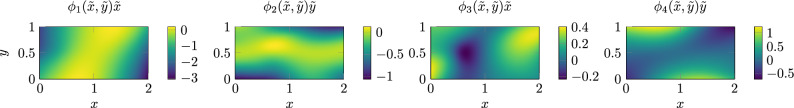
The embedding functions of the TE-QPINN applied to the rescaled coordinates. Certain features of the solution can be recognized.

Table 3 shows the various loss metrics for a different number of qubits. The number of variational layers was chosen to be five. Each configuration was run three times and the results were averaged. As expected, the loss decreases with more qubits. The TE-QPINN with two qubits achieves a lower loss than the Tower-Chebyshev embedding with eight qubits. Figures 8 and 9 show the quantum embedding functions $\Phi (x,y)$ that are used to embed the input coordinate $(x,y)\in \Omega$ into the quantum state.

**Table 3:** Comparison of various loss metrics between the Tower-Chebyshev embedding and TE-QPINN for varying numbers of qubits in the context of the Burgers equation. Five variational layers were employed. The TE-QPINN incorporates 2 hidden layers, each containing 10 neurons.

Qubits	Tower Chebyshev			TE-QPINN		
	Loss	$\text {MSE}_{ref}$	$L_{\infty ,ref}$	Loss	$\text {MSE}_{ref}$	$L_{\infty ,ref}$
2	$3.68\times 10^{1}$	$3.23\times 10^{-2}$	$3.17\times 10^{-1}$	$6.76\times 10^{-2}$	$3.03\times 10^{-5}$	$4.00\times 10^{-2}$
4	5.49	$2.28\times 10^{-3}$	$1.23\times 10^{-1}$	$7.87\times 10^{-3}$	$3.43\times 10^{-6}$	$2.45\times 10^{-2}$
6	$8.87\times 10^{-1}$	$2.05\times 10^{-4}$	$3.31\times 10^{-2}$	$4.48\times 10^{-3}$	$4.60\times 10^{-6}$	$2.08\times 10^{-2}$
8	$3.49\times 10^{-1}$	$1.07\times 10^{-4}$	$2.65\times 10^{-2}$	$1.89\times 10^{-3}$	$4.19\times 10^{-6}$	$1.23\times 10^{-2}$

**Burgers’ equation** The Burgers’ equation is a hyperbolic PDE that develops a strong gradient with increasing time. It can be stated as follows:

38 $$\begin{aligned} \frac{\partial u(t,x)}{\partial t} + u(t,x) \frac{\partial u(t,x)}{\partial x}&= (0.01/\pi ) \frac{\partial ^2u(t,x)}{\partial x^2} \quad t\in [0,0.95], \ x\in [-1,1], \quad u(0,x) = -\sin (\pi x) \end{aligned}$$

The domain is discretized into 50 evenly spaced points in *t* direction and 100 points in *x* direction. The TE-QPINN uses two hidden layers with ten neurons each.

**Fig. 10:**
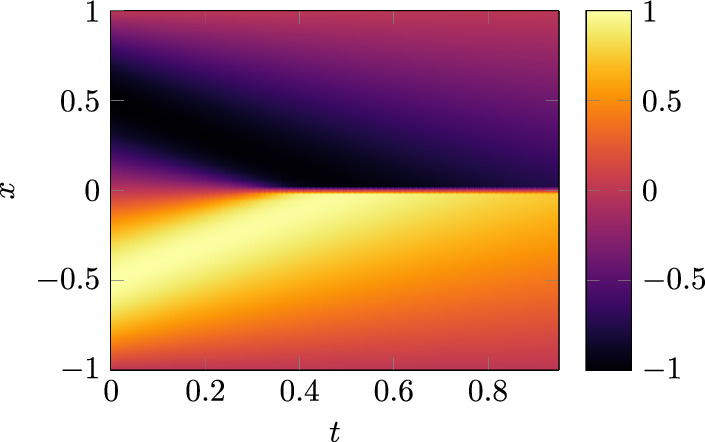
This plot shows the solution of the Burgers equation 38.

**Fig. 11:**
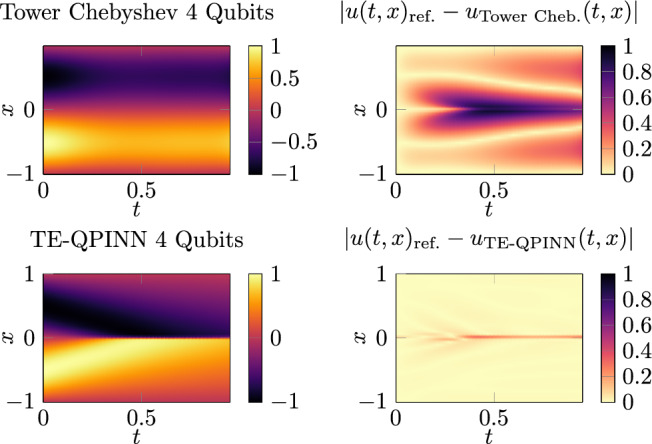
Calculated solutions by the Tower-Chebyshev embedding and the TE-QPINN. Both methods used four qubits and five variational layers.

**Fig. 12:**
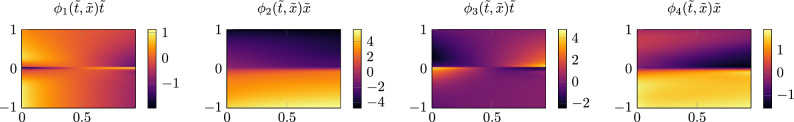
Embedding functions of the TE-QPINN for the Burgers equation.

A plot of the reference solution can be found in Fig. 10 and the computed solution for the Tower-Chebyshev embedding and the TE-QPINN method can be seen in Fig. 11. The Tower-Chebyshev embedding cannot represent the evolving gradient. In contrast, the TE-QPINN has no difficulty with that. The rotation angles of the embedding for the TE-QPINN are shown in Fig. 12. The Tower-Chebyshev embedding uses the same angles as for the Poisson equation shown in Fig. 8.

*Lid-driven cavity* As the final test case, we apply our TE-QPINN approach to the lid-driven cavity. For the 2D case, we can state it as three differential equations; two momentum equations and the mass conservation law:

39 $$\begin{aligned} u\frac{\partial u}{\partial x} + v\frac{\partial u}{\partial y}&= -\frac{1}{\rho }\frac{\partial p}{\partial x} + \nu \left( \frac{\partial ^2 u}{\partial x^2} + \frac{\partial ^2 u}{\partial y^2}\right) \end{aligned}$$

40 $$\begin{aligned} u\frac{\partial v}{\partial x} + v\frac{\partial v}{\partial y}&= - \frac{1}{\rho }\frac{\partial p}{\partial y} + \nu \left( \frac{\partial ^2 v}{\partial x^2} + \frac{\partial ^2 v}{\partial y^2}\right) \end{aligned}$$

41 $$\begin{aligned} \frac{\partial u}{\partial x} + \frac{\partial y}{\partial y}&= 0 \end{aligned}$$

We can reformulate this problem as done in^20^ to include a stream function $\psi$ and compute the velocities as the gradients of $\psi$. This way, the mass conservation law is always fulfilled.

42 $$\begin{aligned} u = - \frac{\partial \psi }{\partial y} \quad v = \frac{\partial \psi }{\partial x} \end{aligned}$$

The lid-driven cavity problem requires the solution of the Navier-Stokes equations on a 2D square $\Omega = [0,1]^2$, where the left, lower and right sides have a nonslip condition. The top boundary is moving to the right with velocity one. The parameters of the equations were set to $\nu =0.1$ and $\rho =1$ such that the Reynolds number is 10. Since pressure in incompressible regimes is determined relative to a constant reference pressure, the pressure at point (0,0) is set to zero. The domain is discretized into 41 equally spaced points in both the x and y direction. A loss scaling factor of 10 is applied to all boundaries. In this example, the two fields $\psi$ and *p* are unknown, therefore two TE-QPINNs are utilized. Each TE-QPINN consists of four qubits and five variational layers. The FNN basis consists of three hidden layers with ten neurons each. The reference solution is computed using OpenFoam with the simpleFoam solver on a 41 by 41 grid.

**Fig. 13:**
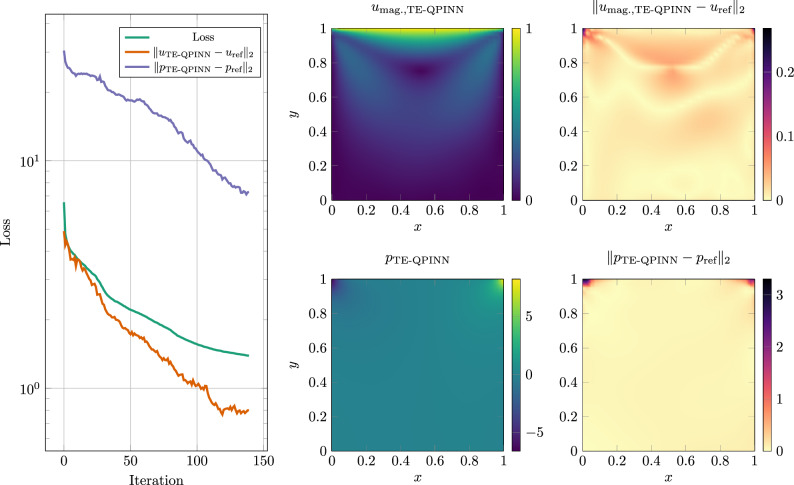
Result of the lid-driven cavity test case. The left plot shows the loss, as well as the $L_2$ norm between the reference solution and the calculated results for each training iteration until convergence. The middle column shows the calculated velocity magnitude, as well as the calculated pressure. The right column shows the $L_2$ norm between the calculated and reference values.

Figure 13 shows the result of the training. The TE-QPINN solution is approaching the reference solution and capturing most of the solution. The main difference between the computed solution and the reference solution is at the two top corners.

### Comparison with PINNs

Finally, we compare PINNs to TE-QPINNs. Both methods are similar in their approach to solving differential equations. The difference is the model used to approximate the function. PINNs use neural networks, and TE-QPINNs use a hybrid system of FNN and PQC. In order to objectively compare both methods, the same amount of trainable parameters (or close to the same amount) will be used to train on the Burgers’ and Poisson problems introduced earlier. The more suitable method would have a lower loss than the other. Thus, a TE-QPINN with four qubits and five variational layers and a FNN with two hidden layers with ten neurons each is compared to two PINN configurations. The first has three hidden layers with ten neurons, and the second has two hidden layers with thirteen neurons. This TE-QPINN has 60 variational parameters in the PQC and 164 FNN parameters, in total 244 trainable parameters. The first PINN configuration has 261 trainable parameters, and the second one has 235.

**Fig. 14:**
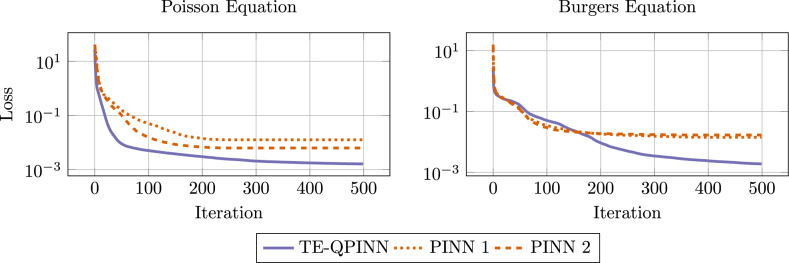
Loss histories of the TE-QPINN and PINN configuration for both the Poisson and Burgers equation.

The losses for both the Poisson and Burgers’ equations are analyzed as previously detailed. The experimental conditions, including discretization points and boundary scaling, followed the benchmarks’ setup as detailed in the previous section. Each configuration was tested with three different initial parameters, after which the results were averaged. Figure 14 presents the findings. In both scenarios, TE-QPINN achieved a lower final loss. Specifically, for the Poisson equation, TE-QPINNs not only converged faster but also achieved a loss almost an order of magnitude lower compared to the best PINN setup. For the Burger equation, TE-QPINNs converged slightly slower; nonetheless, it still outperformed the PINN model by an order of magnitude in terms of final loss.

This simple comparison showed the possible performance improvements of the TE-QPINNs. While we tested the simplest PINN configuration, more sophisticated methods have been proposed^4,21^. However, the embedding FNN used in TE-QPINNs could also benefit from those improvements.

## Discussion

This paper introduced and demonstrated the effectiveness of TE-QPINNs. Building on the concept of differential quantum circuits^11^, TE-QPINNs extend this approach to make it more versatile for a wider range of problems. Importantly, they significantly reduce the number of required qubits, which is particularly advantageous in the era of NISQ devices, where hardware limitations pose a significant challenge. The added computational overhead from this quantum framework is minimal, making it an attractive option in terms of both feasibility and performance.

One key contribution of this work is the use of small FFNs as quantum feature maps. This approach enables efficient computation of gradients on classical hardware, thereby reducing the number of quantum circuit evaluations required. Given that each evaluation on current quantum hardware is both computationally expensive and noisy, this poses a substantial benefit. An idea to consider is mixing different quantum feature maps. Some qubits could be encoded using the Tower-Chebyshev embedding, others with the FNN quantum feature map.

TE-QPINNs have demonstrated strong performance in solving PDEs such as the Poisson and Burgers equations, providing accurate results. Additionally, they offer promising approximations for more complex equations, such as the Navier-Stokes equations. In direct comparison with classical PINNs, TE-QPINNs showed an ability to achieve superior results while using the same number of parameters, highlighting their potential for more efficient optimization in high-dimensional parameter spaces — a known challenge for PINNs. By leveraging the advantages of quantum computing, such as polynomial scaling in exponentially large Hilbert spaces, TE-QPINNs may reduce the number of parameters needed for accurate solutions, which could be transformative for future applications.

This work opens several directions for future research. A stronger theoretical foundation is needed, including error bounds, convergence analysis, and conditions for quantum advantage. Practical feasibility on current quantum hardware should also be explored, considering qubit limitations and noise. Using real hardware, we should be able to asymptotically approximate a point of a possible quantum advantage.

Overall, the results of this research suggest that TE-QPINNs are a promising new direction that motivates further exploration. Their ability to combine quantum computing’s scaling advantages with classical deep learning techniques opens new avenues for solving complex physical problems more efficiently, offering a glimpse into the future of hybrid quantum-classical computation.

## Data Availability

All relevant data and code used in generating the results for this paper can be found in the following Git repository: https://git.rwth-aachen.de/berger.st.11.11/te-qpinns#.

## References

[1] Goodfellow, I., Bengio, Y. & Courville, A. *Deep Learning* (MIT Press, 2016). http://www.deeplearningbook.org.

[2] Shinde, P. P. & Shah, S. A Review of Machine Learning and Deep Learning Applications. In *2018 Fourth International Conference on Computing Communication Control and Automation (ICCUBEA)*, 1–6, 10.1109/ICCUBEA.2018.8697857 (2018).

[3] Raissi, M., Perdikaris, P. & Karniadakis, G. E. Physics-informed neural networks: A deep learning framework for solving forward and inverse problems involving nonlinear partial differential equations. *J. Comput. Phys.* **378**, 686–707. 10.1016/j.jcp.2018.10.045 (2019).

[4] Cuomo, S. et al. Scientific machine learning through physics-informed neural networks: Where we are and what’s next. *J. Sci. Comput.* **92**, 88. 10.1007/s10915-022-01939-z (2022).

[5] Toscano, J. D. *et al.* From pinns to pikans: Recent advances in physics-informed machine learning. arXiv:2410.13228 (2024). 10.1007/s44379-025-00015-1 PMC13290273 42344579

[6] Shor, P. W. Polynomial-time algorithms for prime factorization and discrete logarithms on a quantum computer. *SIAM Rev.* **41**, 303–332 (1999).

[7] Bharti, K. et al. Noisy intermediate-scale quantum algorithms. *Rev. Mod. Phys.* **94**, 015004 (2022).

[8] Cerezo, M. et al. Variational quantum algorithms. *Nat. Rev. Phys.* **3**, 625–644. 10.1038/s42254-021-00348-9 (2021) ArXiv:2012.09265 [quant-ph, stat].

[9] Schuld, M., Sweke, R. & Meyer, J. J. The effect of data encoding on the expressive power of variational quantum machine learning models. *Phys. Rev. A* **103**, 032430. 10.1103/PhysRevA.103.032430 (2021) ArXiv:2008.08605 [quant-ph, stat].

[10] Paine, A. E., Elfving, V. E. & Kyriienko, O. Quantum kernel methods for solving differential equations. *Phys. Rev. A* **107**, 032428. 10.1103/PhysRevA.107.032428 (2023) ArXiv:2203.08884 [cond-mat, physics:quant-ph].

[11] Kyriienko, O., Paine, A. E. & Elfving, V. E. Solving nonlinear differential equations with differentiable quantum circuits. *Phys. Rev. A* **103**, 052416. 10.1103/PhysRevA.103.052416 (2021) ArXiv:2011.10395 [cond-mat, physics:quant-ph].

[12] Schuld, M., Bergholm, V., Gogolin, C., Izaac, J. & Killoran, N. Evaluating analytic gradients on quantum hardware. *Phys. Rev. A* **99**, 032331. 10.1103/PhysRevA.99.032331 (2019).

[13] Crooks, G. E. Gradients of parameterized quantum gates using the parameter-shift rule and gate decomposition. ArXiv:1905.13311 [quant-ph] (2019).

[14] Mari, A., Bromley, T. R. & Killoran, N. Estimating the gradient and higher-order derivatives on quantum hardware. *Phys. Rev. A* **103**, 012405. 10.1103/PhysRevA.103.012405 (2021).

[15] Liu, D. C. & Nocedal, J. On the limited memory BFGS method for large scale optimization. *Math. Program.* **45**, 503–528. 10.1007/BF01589116 (1989).

[16] Cerezo, M., Sone, A., Volkoff, T., Cincio, L. & Coles, P. J. Cost function dependent barren plateaus in shallow parametrized quantum circuits. *Nat. Commun.* **12**, 1791. 10.1038/s41467-021-21728-w (2021). 33741913 10.1038/s41467-021-21728-w PMC7979934

[17] Bergholm, V. *et al.* PennyLane: Automatic differentiation of hybrid quantum-classical computations. ArXiv:1811.04968 [physics, physics:quant-ph] (2022).

[18] Paszke, A. *et al.* Pytorch: An imperative style, high-performance deep learning library. *CoRR* arXiv:abs/1912.01703 (2019).

[19] Project, F. Poisson equation docs.fenicsproject.org. https://docs.fenicsproject.org/dolfinx/main/python/demos/demo_poisson.html. (Accessed 18 Oct 2024).

[20] Raissi, M., Perdikaris, P. & Karniadakis, G. E. Physics Informed Deep Learning (Part II): Data-driven Discovery of Nonlinear Partial Differential Equations. ArXiv:1711.10566 [cs, math, stat] (2017).

[21] Wang, S., Wang, H. & Perdikaris, P. On the eigenvector bias of Fourier feature networks: From regression to solving multi-scale PDEs with physics-informed neural networks. *Comput. Methods Appl. Mech. Eng.* **384**, 113938. 10.1016/j.cma.2021.113938 (2021).

